# Single-cell ionic current phenotyping explains stem cell-derived cardiomyocyte action potential morphology

**DOI:** 10.1152/ajpheart.00063.2024

**Published:** 2024-03-15

**Authors:** Alexander P. Clark, Siyu Wei, Kristin Fullerton, Trine Krogh-Madsen, David J. Christini

**Affiliations:** ^1^Department of Biomedical Engineering, Cornell University, Ithaca, New York, United States; ^2^Department of Physiology and Pharmacology, SUNY Downstate Health Sciences University, Brooklyn, New York, United States; ^3^Department of Physiology and Biophysics, Weill Cornell Medicine, New York, New York, United States; ^4^Institute for Computational Biomedicine, Weill Cornell Medicine, New York, New York, United States

**Keywords:** arrhythmias, computer simulation, induced pluripotent stem cells, iPSC-CMs, patch clamp

## Abstract

Human induced pluripotent stem cell-derived cardiomyocytes (iPSC-CMs) are a promising tool to study arrhythmia-related factors, but the variability of action potential (AP) recordings from these cells limits their use as an in vitro model. In this study, we use recently published brief (10 s), dynamic voltage-clamp (VC) data to provide mechanistic insights into the ionic currents contributing to AP heterogeneity; we call this approach rapid ionic current phenotyping (RICP). Features of this VC data were correlated to AP recordings from the same cells, and we used computational models to generate mechanistic insights into cellular heterogeneity. This analysis uncovered several interesting links between AP morphology and ionic current density: both L-type calcium and sodium currents contribute to upstroke velocity, rapid delayed rectifier K^+^ current is the main determinant of the maximal diastolic potential, and an outward current in the activation range of slow delayed rectifier K^+^ is the main determinant of AP duration. Our analysis also identified an outward current in several cells at 6 mV that is not reproduced by iPSC-CM mathematical models but contributes to determining AP duration. RICP can be used to explain how cell-to-cell variability in ionic currents gives rise to AP heterogeneity. Because of its brief duration (10 s) and ease of data interpretation, we recommend the use of RICP for single-cell patch-clamp experiments that include the acquisition of APs.

**NEW & NOTEWORTHY** We present rapid ionic current phenotyping (RICP), a current quantification approach based on an optimized voltage-clamp protocol. The method captures a rich snapshot of the ionic current dynamics, providing quantitative information about multiple currents (e.g., *I*_Ca,L_, *I*_Kr_) in the same cell. The protocol helped to identify key ionic determinants of cellular action potential heterogeneity in iPSC-CMs. This included unexpected results, such as the critical role of *I*_Kr_ in establishing the maximum diastolic potential.

## INTRODUCTION

Human induced pluripotent stem cell-derived cardiomyocytes (iPSC-CMs) are a promising model to study congenital and acquired cardiac arrhythmias. The depth of insights from electrophysiological studies with these cells is limited by their immature phenotype and cell-to-cell variability (1). There is substantial laboratory-to-laboratory heterogeneity in the action potential (AP) morphology of iPSC-CMs, and even genetically identical iPSC-CMs derived from the same donor display significant variability (2, 3). Such shortcomings make it difficult to glean meaningful physiological information from patient-specific iPSC-CMs (4) and have led to inconsistent results in multisite drug cardiotoxicity screening studies (5). Developing an understanding of iPSC-CM heterogeneity is an essential step to improve the utility of these cells as a tool for use in precision medicine.

Here, we present a new method called rapid ionic current phenotyping (RICP) that we use to study variations in the ionic currents contributing to iPSC-CM AP heterogeneity. In this study, we use recently published iPSC-CM patch-clamp data from our laboratory (6), including short (10 s) voltage-clamp (VC) recordings that provide insight into the presence and relative size of several key cardiac ionic currents. The novelty of this study is in the use of such a brief VC protocol to draw the following set of conclusions about the iPSC-CMs used here:

•  L-type calcium current (*I*_Ca,L_) drives upstroke in iPSC-CMs with a depolarized AP morphology.
•  Rapid delayed rectifier K^+^ current (*I*_Kr_) plays a role in establishing the maximal diastolic potential.
•  Seal-leak current contaminates VC recordings and contributes to a depolarized maximal diastolic potential.
•  Large positive currents present at potentials >40 mV correlate with a shortened AP duration.
•  Several cells contain a strong outward current at 6 mV that is not present in computational AP models and correlates with AP duration.

## METHODS

### iPSC-CM Cell Culture and Electrophysiological Setup

The in vitro data were previously published (6).

Frozen iPSC-CMs were thawed from five different vials that were purchased from the Stanford Cardiovascular Institute Biobank. Before patch-clamp recording, each cell was plated on a coverslip and had been independently cultured for 3–13 days in a 24-well plate. These cells were derived from an African-American female donor in a process approved by Stanford University Human Subjects Research Institutional Review Board.

Cells were prepared for electrophysiological experiments following the steps described in Ref. 6. Briefly, cells were thawed and cultured as a monolayer in one well of a six-well plate precoated with 1% Matrigel. Cells were cultured with RPMI media (Fisher/Corning 10–040-CM) containing 5% FBS and 2% B27 and kept in an incubator at 37°C, 5% CO_2_, and 85% humidity. After 48 h, cells were lifted with 1-mL Accutase, diluted to 100,000 cells/mL, and replated on 124-sterile, 8-mm coverslips precoated with 1% Matrigel. Cells were cultured with RPMI media that was swapped every 48 h. Cells were patched between *days 5* and *15* after thaw.

Voltage-clamp and current-clamp recordings were acquired from 40 cells using the perforated patch technique and an amplifier equipped with a voltage follower circuit (Model 2400; A-M Systems, Sequim, WA). We excluded one cell from the analyses in this study because it had spontaneous alternans with inconsistent AP features. All cells had a prerupture seal of >300 MΩ.

### Voltage-Clamp Protocol

We have previously developed a voltage-clamp protocol consisting of multiple short segments, each designed to isolate one key ionic current (6). The protocol was designed using optimization techniques and a mathematical model of iPSC-CMs (7) to maximize, one at a time, the contribution to total current by each of seven key currents: *I*_Kr_, *I*_Ca,L_, sodium current (*I*_Na_), transient outward K^+^ current (*I*_to_), inward rectifier K^+^ current (*I*_K1_), funny current (*I*_f_), and slow delayed rectifier K^+^ current (*I*_Ks_).

During our analysis, we found that the current measured 100 ms after a depolarizing step to 6 mV (*I*_6mV_) was substantially different in many cells from that predicted by the mathematical model and we therefore included *I*_6mV_ as an 8th current measure. For each current-isolating segment, we quantified the recorded total current *I*_out_ using either the minimum or the average over a 2 ms span centered at the following values: *I*_6mV_ (600 ms, average), *I*_Kr_ (1,262 ms, average), *I*_Ca,L_ (1,986 ms, minimum), *I*_Na_ (2,760 ms, minimum), *I*_to_ (3,641 ms, average), *I*_K1_ (4,300 ms, average), *I*_f_ (5,840 ms, average), and *I*_Ks_ (9,040 ms, average).

### AP Feature Calculations

The transmembrane potential of each cell was recorded for 10 s. Of the 39 cells, 12 were not spontaneously beating. We computed a minimal potential (MP) for these nonspontaneous cells.

For cells that were spontaneously beating, we computed their MP (in this case, the minimum voltage during the AP), action potential duration at 90% repolarization (APD_90_), cycle length (CL), and maximal upstroke velocity (d*V*/d*t*_max_). The average of each feature was calculated for all cells that produced more than one AP during the 10-s recording.

### iPSC-CM Mathematical Models

For comparison and to guide the analysis of our experimental data, we used two different mathematical models of iPSC-CM electrophysiology: the Paci et al. model (8) and the Kernik et al. model (7). We set the cell capacitance (*C*_m_) of these models to 45 pF, which is centrally located in the range (18–98 pF) of the capacitances for cells used in this study. To avoid long transients and to better simulate our perforated patch experimental setup, we fixed intracellular sodium and potassium concentrations ([Na^+^]_i_ and [K^+^]_i_) to their baseline steady-state values (taken after 1,000 s of spontaneous or paced current-clamp simulation). Because the leak through the imperfect pipette-membrane seal during single-cell patch-clamp experiments can substantially impact the electrophysiological recordings in these cells, we included a linear leak current (*I*_leak_) in the mathematical models (3). We used a baseline value of 2 GΩ for the seal resistance.

In addition to the seal-leak current, for voltage-clamp simulations, we included explicit modeling of the following experimental artifacts: liquid junction potential offset (−2.8 mV), access resistance (20 MΩ), and series resistance (*R*_s_) compensation (70%), including supercharging (9, 10).

### Population of Models and Sensitivity Analysis

A population of 500 individuals with unique parameters sets was generated using both the Paci and Kernik models with experimental artifact equations by randomly sampling conductances of *I*_Na_, *I*_Ca,L_, *I*_Kr_, *I*_Ks_, *I*_to_, *I*_K1_, *I*_f_, *I*_leak_, sodium-calcium exchange current (*I*_Na,Ca_), sodium potassium pump current (*I*_Na,K_), sodium background current (*I*_b,Na_), calcium background current (*I*_b,Ca_), as well as *C*_m_ and *R*_s_ from a log uniform distribution between 0.25 and four times their baseline values. Of the 500 Paci models, 18 were excluded because of numerical integration issues. The Kernik and Paci populations produced 150 and 194 spontaneously beating individuals, respectively. All individuals (whether spontaneously beating or not) were used to calculate the MP. Only spontaneously beating individuals were used to calculate d*V*/d*t*_max_, APD_90_, and CL. We used a Spearman correlation to determine the sensitivity of the current-isolating time points to these parameters.

### Linear Regression

A linear least-squares regression was used to compare ionic currents and AP features for both the in vitro and the in silico data. A Spearman correlation coefficient and *P* value were calculated for these data.

### Software and Simulations

Simulations were performed in Myokit v1.33.7 (11). Additional analysis was done in Python using NumPy v1.21.6 and SciPy v1.7.3 (12). All data, code, and models can be accessed from GitHub (https://github.com/Christini-Lab/ap-vc-correlations.git).

## RESULTS

### iPSC-CMs Are Heterogeneous

**Figure 1.**
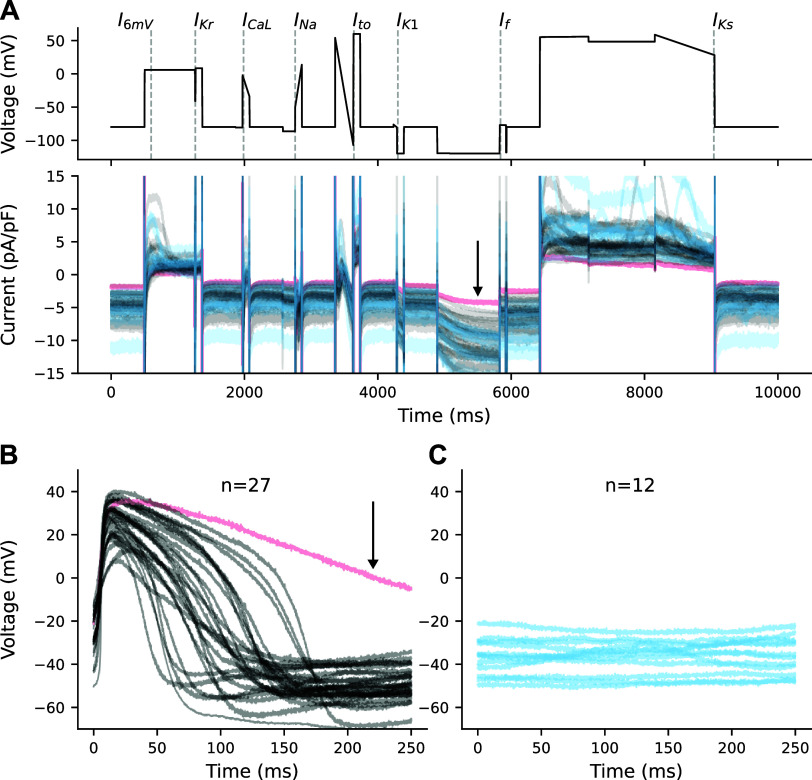
Voltage-clamp (VC) variations and membrane potential in 39 cells. *A*: optimized voltage-clamp protocol (*top*) with lines overlaid that indicate time points designed to isolate each current. Responses to the voltage-clamp protocol (*bottom*) for spontaneous (black), quiescent (blue), and the outlier (pink). *B*: action potentials (APs) from spontaneously beating cells (*n* = 27). Pink AP is an outlier with an AP duration at 90% repolarization (APD_90_) >2× longer than the rest of the population. *C*: voltage recordings from quiescent and depolarized cells (*n* = 12).

We used data from 39 perforated patch-clamped cells (6): 27 were spontaneously beating, and 12 were quiescent and depolarized (Fig. 1, *B* and *C*). There is substantial cell-to-cell heterogeneity. Reporting the variation as the standard deviation (SD), we calculate a minimum potential (MP) of −52 ± 10 mV, action potential duration at 90% repolarization (APD_90_) of 127 ± 70 ms, and maximum upstroke velocity (d*V*/d*t*_max_) of 10.5 ± 6.6 V/s.

Within the subset of spontaneously beating cells, there is additional heterogeneity in that some have a predictable and consistent cycle length (CL), while the CL of others is highly variable (Supplemental Fig. S1; all Supplemental Figures are available at https://doi.org/10.6084/m9.figshare.25289914.v3). This level of heterogeneity is consistent with variations observed in other iPSC-CM electrophysiology studies (18, 22).

### Rapid Ionic Current Phenotyping Provides Insight into AP Outliers

As detailed in the methods, we recently published a voltage-clamp protocol (6) that was designed to maximize the isolation for each of the following seven key ionic currents: *I*_Kr_, *I*_Ca,L_, *I*_Na_, *I*_to_, *I*_K1_, *I*_f_, and *I*_Ks_. The protocol works by stepping to voltages designed to maximize the contribution of each current at different time points. The target current (e.g., *I*_Kr_) during an isolating segment, however, can be contaminated with other ionic (e.g., *I*_Na,K_) or artifact (*I*_leak_) currents. Yet, we show here and in our previous study (6) that we can draw conclusions about target currents based on the information gleaned from these isolating segments. Because of its short duration (10 s) and design to target multiple currents, we are calling this approach rapid ionic current phenotyping (RICP).

Figure 1*A* shows the voltage-clamp protocol (*top*) and heterogeneity in ionic current responses (*bottom*) from the 39 cells included in this study. On the voltage-clamp protocol, we have overlaid dashed lines to highlight current-isolating time points that we use to study ionic current dynamics. In addition to the seven designed current-isolating segments, we included another time point measure (*I*_6mV_, the current recorded 100 ms after a depolarizing step to 6 mV), as we found that it provides insight into dynamics that are not present during other portions of the voltage-clamp protocol.

Spontaneously beating cells are plotted in black and quiescent cells are in blue (Fig. 1, *A*–C). The pink spontaneous cell is an outlier, with an APD_90_ value greater than two times longer than the rest of the population. When compared with the rest of the population, the outlier cell appears to have the smallest total current during the regions of the protocol designed to isolate *I*_K1_, *I*_f_, and *I*_Ks_ (Supplemental Fig. S2).

### RICP Identifies *I*_ca,L_ as Driver of Upstroke in Depolarized Cells

**Figure 2.**
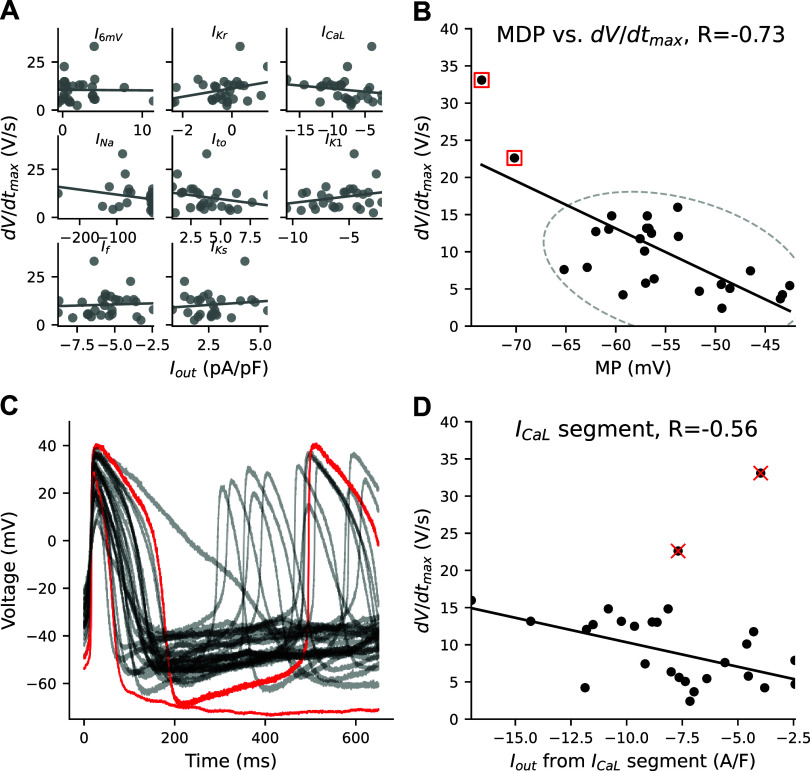
*I*_Ca,L_ helps drive upstroke in depolarized human induced pluripotent stem cell-derived cardiomyocytes (iPSC-CMs). *A*: there is no correlation between *I*_out_ from the eight current-isolating segments and maximal upstroke velocity (d*V*/d*t*_max_). *B*: d*V*/d*t*_max_ decreases as minimal potential (MP) depolarizes. Red boxes denote the two cells with the most hyperpolarized MP and largest d*V*/d*t*_max_. *C*: two highlighted cells from *B* share few action potential (AP) commonalities with one another other than their relatively hyperpolarized MP and large d*V*/d*t*_max_. *D*: a trend emerges between *I*_out_ during the *I*_Ca,L_-isolating segment and d*V*/d*t*_max_ when the two hyperpolarized cells are removed from the analysis. *I*_Ca,L_, L-type calcium current.

Figure 2*A* displays the relationship between the total current during each current-isolating segment and the d*V*/d*t*_max_ for all cells. None of the eight current-isolating segments correlate with d*V*/d*t*_max_. Figure 2*A* shows the likely presence of *I*_Na_ in many of these cells: we draw this conclusion because *I*_Na_ is the only current expected to generate a total recorded current (*I*_out_) of < −40 A/F within 2 ms after the *I*_Na_ voltage step. However, the upstroke velocity is small in most cells and does not correlate with the current measured during the *I*_Na_-isolating segment. The small upstroke velocities and the lack of correlation with *I*_Na_ was an unexpected finding, given that *I*_Na_ is present in many of these cells.

Although there is no significant relationship with the VC segments, d*V*/d*t*_max_ does correlate with MP (Fig. 2*B*); d*V*/d*t*_max_ increases as MP becomes more hyperpolarized. The two cells with MP below −70 mV (denoted with red squares) stand out as having much larger upstroke velocities than the rest of the population. These two cells likely repolarize enough to make some sodium channels available for an *I*_Na_-driven upstroke. Other than having relatively hyperpolarized MP values, these two cells produce APs with few morphological similarities (Fig. 2*C*).

Interestingly, when we exclude these cells from the regression analysis, and only consider iPSC-CMs with MP > −70 mV, a significant relationship emerges between the *I*_Ca,L_-isolating segment and d*V*/d*t*_max_ (Fig. 2*D*). This indicates that *I*_Ca,L_ may be, at least partly, responsible for the upstroke in cells with MP > −70 mV.

### RICP Identifies *I*_Kr_-Isolating Segment as Predictor of MP

**Figure 3.**
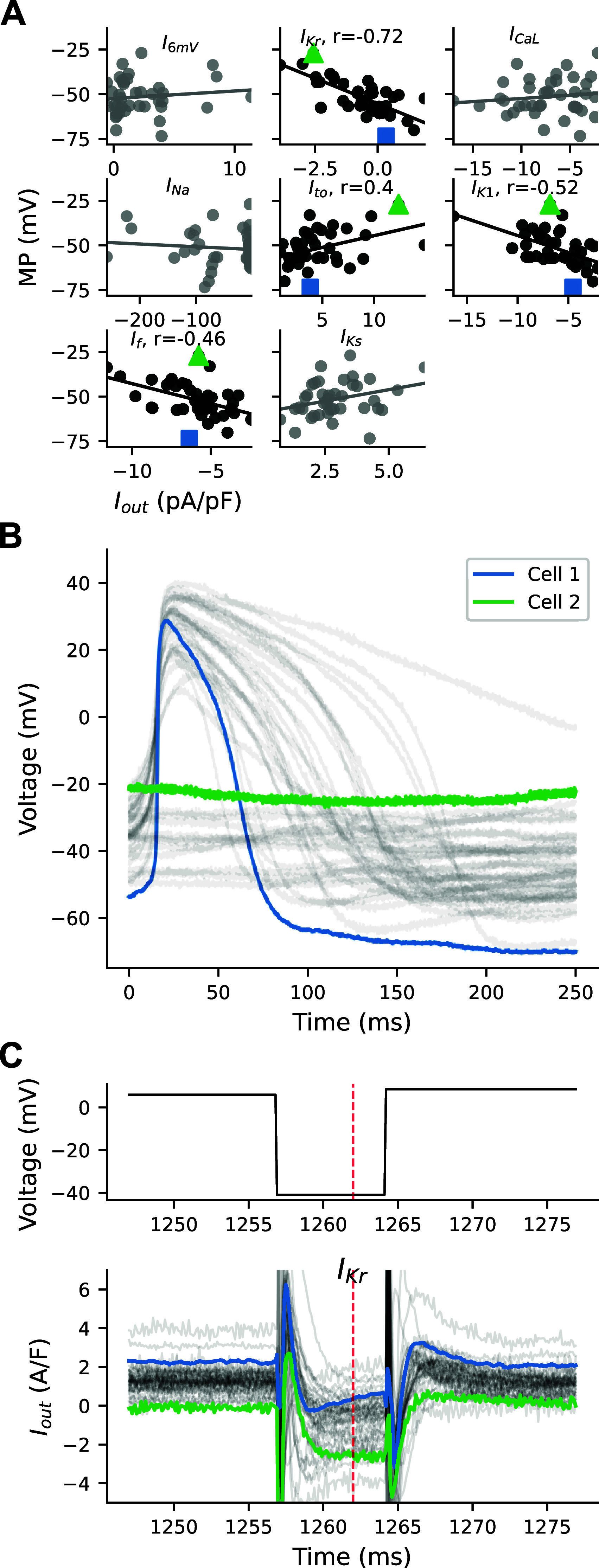
Correlations between ionic current segments and minimal potential (MP). *A*: *I*_out_ from the *I*_Kr_, *I*_to_, *I*_K1_, *I*_f_, and *I*_Ks_ segments correlate significantly (*P* < 0.05) with MP. *B*: current-clamp recordings from all cells. The human induced pluripotent stem cell-derived cardiomyocytes (iPSC-CMs) with the most hyperpolarized (blue, *cell 1*) and depolarized (green, *cell 2*) MP values are compared. *C*: traces from the *I*_Kr_-isolating segment of the voltage-clamp (VC) protocol for all cells. VC protocol (*top*) includes a 750-ms prestep at 6 mV, followed by a 7-ms step at −41 mV, and then a step to 9 mV. VC protocol was designed to isolate *I*_Kr_ at 1,262 ms (red dashed line). *Cell 1* (blue) and *cell 2* (green) are highlighted. *I*_f_, funny current; *I*_Kr_, rapid delayed rectifier K^+^ current; *I*_K1_, inward rectifier K^+^ current; *I*_Ks_, slow delayed rectifier K^+^ current; *I*_out_, total recorded current; *I*_to_, transient outward K^+^ current.

Four current-isolating segments (*I*_Kr_, *I*_to_, *I*_K1_, and *I*_f_) of the VC protocol correlate with MP (Fig. 3*A*), with the *I*_Kr_-isolating segment being the strongest correlate (*R* = 0.72). We selected cells at the two MP extremes to illustrate the relationship between the *I*_Kr_-isolating segment and MP (Fig. 3*B*): *cell 1* (blue) is the most hyperpolarized in the population (MP = −73 mV and APD_90_ = 81 ms), and *cell 2* is the most depolarized (quiescent with MP of −27 mV).

A plot of the *I*_Kr_-isolating section, which was previously designed and subsequently validated with drug studies (see Fig. 5 from Ref. 6 for details), shows the voltage command (*top*) and current responses (*bottom*) for all cells in the population (Fig. 3*C*). During this section of the protocol, cells were clamped to 6 mV for 750 ms, then stepped to −41 mV for 7 ms, and then back up to 9 mV. At the time point designed to isolate *I*_Kr_ (red dashed line), *cell 1* conducts a small positive total current (0.3 A/F) that increases in size, which is consistent with *I*_Kr_ recovering from rectification. In contrast, *cell 2* conducts a net negative current (−2.5 A/F) and does not show *I*_Kr_ recovery characteristics, suggesting that this cell has much less *I*_Kr_. These cells are examples of the population-level correlation seen between the *I*_Kr_-isolating segment and MP. They indicate that *I*_Kr_, and other currents that may be present during the step to −40 mV, likely play a critical role in establishing MP in these cells.

We used an iPSC-CM mathematical model with a linear seal-leak current (see methods) to further study the potential role of *I*_Kr_ in establishing an MP. Supplemental Fig. S3 shows the effect of scaling *I*_Kr_ conductance (*g*_Kr_) on the MP for a model with a 2 GΩ seal resistance. While holding all other parameters constant, reducing *g*_Kr_ by >70% of baseline results in substantial depolarization. The 10–90% range of our experimental data corresponds to a roughly 70–85% reduction in *g*_Kr_ (Supplemental Fig. S3*B*). These model findings are consistent with the correlations seen between the experimental *I*_Kr_-isolating *I*_out_ measurements and MP.

### Seal-Leak Current Likely Contributes to the Depolarized MP

In addition to the *I*_Kr_-isolating segment, the *I*_to_, *I*_K1_, *I*_f_, and *I*_Ks_-isolating segments also weakly correlate with MP (Fig. 3*A*). Some of these correlations are unexpected and counterintuitive as, e.g., *I*_to_ is typically inactivated during phases 3 and 4 of the AP. We hypothesized that these correlations may be influenced by a leak artifact current caused by an imperfect seal between the pipette tip and cell membrane; we recently demonstrated that this leak current can substantially depolarize the MP of iPSC-CMs (3).

To investigate the role of *I*_leak_ on these segments, we developed a population of computational models that include patch-clamp experimental artifact equations (Supplemental Fig. S4). In this in silico population, the *I*_Kr_ segment is also the main correlate with MP, and *I*_to_, *I*_Ks_, and *I*_f_ are more weakly correlated (Supplemental Fig. S6).

A sensitivity analysis of the population of models shows that the segments weakly correlating with MP in the experiments (i.e., the *I*_to_, *I*_K1_, *I*_f_, and *I*_Ks_ segments) are all sensitive to the conductance of *I*_leak_ (i.e., *g*_leak_, Supplemental Fig. S4C). *I*_leak_ is modeled as a linear current with a reversal potential of zero, and so it will increase when cells are clamped to voltages that are far from 0 mV. Seeing as *I*_Ks_ and *I*_to_ are elicited by stepping to large positive voltages and *I*_K1_ and *I*_f_ to large negative voltages, this is consistent with these segments being contaminated by *I*_leak_. Increased *g*_leak_ increases the total (net outward) currents computed at positive voltages (e.g., *I*_Ks_ and *I*_to_ segments) and depolarizes the MP, consistent with the positive correlation seen between MP and the *I*_Ks_ and *I*_to_ segments in our experiments. Similarly, the negative correlations between MP and *I*_K1_ and *I*_f_ in our cells is consistent with the in silico results where increased *g*_leak_ during these hyperpolarized segments in the models contribute to the large (net inward) current and depolarized MP values.

### RICP Identifies Strong Outward Currents as Drivers of APD_90_

**Figure 4.**
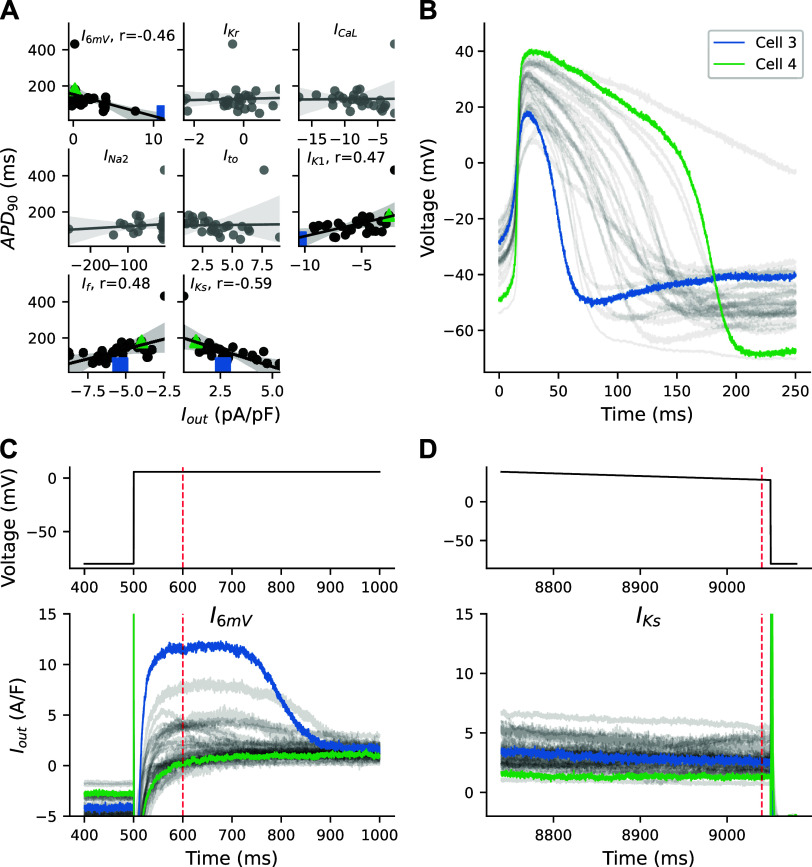
Correlations between ionic currents and action potential (AP) duration at 90% repolarization (APD_90_). *A*: *I*_out_ from the *I*_6mV_, *I*_K1_, *I*_f_, and *I*_Ks_ segments correlate significantly (*P* < 0.05) with APD_90_. *B*: cells that generated the shortest (short) and second longest (long) APs were investigated. Current responses from cells during *I*_6mV_ (*C*) and *I*_Ks_ (*D*) step. *I*_f_, funny current; *I*_K1_, inward rectifier K^+^ current; *I*_Ks_, slow delayed rectifier K^+^ current; *I*_out_, total recorded current.

Four segments (*I*_6mV_, *I*_K1_, *I*_f_, and *I*_Ks_) of the VC protocol correlate with APD_90_ (Fig. 4*A*). Three of the segments (*I*_K1_, *I*_f_, and *I*_Ks_) were designed to isolate potassium-conducting currents, each of which is clamped to voltages far from 0 mV. The *I*_6mV_ segment was added to quantify observed current dynamics different from those produced by the mathematical models and which we hypothesized could be explanatory of AP morphological differences.

To illustrate the relationship between currents and APD_90_, we investigated the current responses from two different cells (Fig. 4*B*): one with the shortest APD_90_ (*cell 3*) and one with the second longest APD_90_ (*cell 4*).

The strong correlation between the *I*_Ks_-isolating segment and APD_90_ indicates that the net outward current at large positive voltages affects APD_90_. *Cell 3* has a slightly larger outward current during this *I*_Ks_-isolating segment when compared with *cell 4* (Fig. 4*D*).

*I*_f_ and *I*_K1_-isolating segments correlate less strongly with APD_90_ and in the opposite direction than what would be expected. Although both currents are expected to influence AP duration, their current-isolating segments are likely contaminated with *I*_leak_ (Supplemental Fig. S4) that is contributing in the opposite direction (3).

The outward current present during the *I*_6mV_ segment is much larger in *cell 1* than any other in the population and is very likely driving the rapid repolarization shown in this cell. In contrast, *cell 2* has a nearly balanced net current during the *I*_6mV_ segment. Comparing our population of models to experimental current responses shows that the experimental responses for many of the cells (11/39) have a strong repolarizing ionic current at 6 mV that is not represented in the iPSC-CM electrophysiological model (Supplemental Fig. S5).

## DISCUSSION

Here, we propose RICP as a novel approach to study the ionic current underpinnings of AP heterogeneity. By analyzing data from a brief, optimized 10-s VC protocol, we generate the following insights about the iPSC CMs used in this study:

•  *I*_Ca,L_ is partly responsible for driving upstroke when MP is >−70 mV.
•  *I*_Kr_ contributes to establishing the MP.
•  *I*_leak_ contaminates VC responses at very positive and negative voltages and contributes to MP.
•  Strong outward current elicited in the range of *I*_Ks_ activation correlates with AP duration.
•  A subset of these cells has an unidentified outward current present at 6 mV that is not recapitulated by iPSC-CM mathematical models.

Overall, this study demonstrates both the large cell-to-cell heterogeneity in iPSC-CMs and provides a tractable method to study the ionic current determinants of such variability during patch-clamp experiments.

### RICP Is a Tool to Understand Ionic Current Mechanisms of iPSC-CM Heterogeneity

We used our recently published VC protocol to capture a rich snapshot of current dynamics that is explanatory of iPSC-CM AP morphology. This 10-s protocol provided information about several currents (e.g., *I*_Ca,L_, *I*_Kr_) and their effects on AP morphology (d*V*/d*t*_max_, MP). Other studies have collected VC data for one or two currents and shown correlations with certain AP morphological features from the same cells (13). However, we believe this is the first study that attempts to collect information about several currents with a <10-s protocol and use these data to explain AP features.

The VC protocol was designed to identify the presence and relative size of seven individual currents, but each of the segments is contaminated, at least to some extent, by off-target currents. For example, *I*_leak_ contaminates every segment of the protocol, as it contributes current at all voltages, with increased *I*_leak_ at voltages far from 0 mV. The sensitivity analysis shows how the *I*_K1_, *I*_to_, *I*_f_, and *I*_Ks_ segments, all of which are clamped far from 0 mV, are sensitive to changes in the seal-leak resistance (Fig. 4*C*). Some currents (e.g., *I*_Na_) have distinct dynamics that provide for relatively easy isolation, but others (e.g., *I*_Kr_) always open when several currents conduct ions.

The isolation of currents could potentially be improved through the development of new VC protocols with different optimization methods or by using new computational models that better capture the dynamics of iPSC-CMs used in a specific experimental context. However, we believe new protocols will only result in marginal improvements. Instead, we think there is far more to gain by developing methods to tease apart contributions from the different currents. To further validate the RICP approach, and depending on the objective of the experiments, one could sequentially dissect current contributions (14) or fit computational models to individual traces (15), either of which could provide estimates of cell-specific ionic current densities. These approaches could be used in conjunction with a leak compensation current [see Clark et al. (3) for details] during current-clamp recordings to probe the independent contributions of seal-leak and ionic-current densities to AP morphology.

### *I*_ca,L_ Likely Drives Upstroke in Many iPSC-CM Studies

Using RICP, we identified *I*_Ca,L_ as a contributor to upstroke velocity in the depolarized iPSC-CMs used in this study. Despite the presence of *I*_Na_ in most of our cells, *I*_Ca,L_ appears to be the predominant driver of upstroke. This is likely because, at depolarized MP values (e.g., >−70 mV), *I*_Na_ is inactive and cannot generate an inward current to drive upstroke.

This implies a mechanism like AP generation in SA nodal cells, despite the presence of *I*_Na_ (16), the upstroke is primarily driven by calcium currents (17). This finding also hints at *I*_Ca,L_ as the likely driver of upstroke in many other studies where MP < −65 mV or d*V*/d*t*_max_ is <20 V/s (18, 19). As we and others have shown, it is possible to recover a polarized MP by dynamically clamping a synthetic *I*_K1_ (6, 20). Such an approach makes it possible for *I*_Na_, among other currents, to recover from inactivation and results in APs with a faster upstroke velocity and more mature appearance.

### *I*_Kr_ Is Likely an Important Current in Establishing MP in Depolarized iPSC-CMs

*I*_K1_ conductance is often thought to be reduced in iPSC-CMs relative to adult cardiomyocytes, resulting in depolarized MP. However, iPSC-CMs can have large amounts of *I*_K1_, and yet, still be depolarized compared with adult cells (18). One hypothesis for this discrepancy in MP between adult and iPSC cardiomyocytes is the increased role of *I*_leak_ in iPSC-CMs (3, 18) caused by their smaller size relative to adult cardiomyocytes. In this study, we identify *I*_Kr_ as likely playing a role in establishing the MP of iPSC-CMs. The correlation between *I*_Kr_ and MP is unlikely to be substantially influenced by leak as the VC step to isolate *I*_Kr_ is close to the expected seal leak reversal potential.

In both our experimental data and in populations of models based on either the Paci or Kernik models, the *I*_Kr_-isolating segment has the strongest correlation to MP (−0.66, −0.54, and −0.58 for experimental data, Paci, and Kernik, respectively, Fig. 3 and Supplemental Figs. S6 and S7).

This finding agrees with previous work showing that iPSC-CM MP values are sensitive to E-4031 (21). Our modeling work indicates that with a small *I*_leak_ (2-GΩ seal) and substantial reduction in baseline *I*_Kr_, the model will depolarize to MP values like those shown in our study.

### Limitations and Future Directions

The RICP method shows how a brief VC protocol can be used to provide mechanistic insights into AP morphology and heterogeneity. This protocol, however, does not perfectly isolate each of the seven currents, and so the causal relationships of currents with AP morphological features are likely weaker than if we conducted traditional drug block experiments. This is the tradeoff we make in attempting to collect data for seven different ion channels all in the same cell. In the future, we believe methods that tease apart the current contributions at each time point have the potential to improve insights drawn from this VC protocol, such as ion channel coexpression patterns. Furthermore, we were unable to deduce the identity of the outward current at 6 mV, given the voltage and nonlinearity in the current’s response, we hypothesize that this is a potassium-conducting species with a time or state (perhaps concentration) dependence, but further drug studies would be required to definitively identify this current.

In this study, we focus on a set of cells derived from a single individual, using a single differentiation method, within the perforated patch-clamp experimental context used in our laboratory. Although we expect the RICP approach to generalize to other cell lines and contexts, the iPSC-CM characterization here (e.g., *I*_Ca,L_ driving upstroke) may not be descriptive of similar cells in other studies. In the future, it would be interesting to conduct this RICP approach on cells from multiple donors, across multiple differentiation batches, and in multiple laboratory settings.

## DATA AVAILABILITY

All data can be accessed or generated from the GH page: https://github.com/Christini-Lab/ap-vc-correlations.

## SUPPLEMENTAL DATA

10.6084/m9.figshare.25289914.v3 Supplemental Figs. S1–S7: https://doi.org/10.6084/m9.figshare.25289914.v3.

## GRANTS

This work was supported by National Heart, Lung, and Blood Institute Grants U01HL136297 (to D.J.C.) and F31HL154655 (to A.P.C.).

## DISCLOSURES

No conflicts of interest, financial or otherwise, are declared by the authors.

## AUTHOR CONTRIBUTIONS

A.P.C., S.W., K.F., T.K-M., and D.J.C. conceived and designed research; A.P.C. and S.W. performed experiments; A.P.C. and K.F. analyzed data; A.P.C., S.W., K.F., T.K-M., and D.J.C. interpreted results of experiments; A.P.C. and K.F. prepared figures; A.P.C., T.K-M., and D.J.C. drafted manuscript; A.P.C., S.W., K.F., T.K-M., and D.J.C. edited and revised manuscript; A.P.C., S.W., K.F., T.K-M., and D.J.C. approved final version of manuscript.

## References

[1] Goversen B, van der Heyden MA, van Veen TA, de Boer TP. The immature electrophysiological phenotype of iPSC-CMs still hampers in vitro drug screening: special focus on I_K1_. Pharmacol Ther 183: 127–136, 2018. doi:10.1016/j.pharmthera.2017.10.001. 28986101

[2] Ma J, Guo L, Fiene SJ, Anson BD, Thomson JA, Kamp TJ, Kolaja KL, Swanson BJ, January CT. High purity human-induced pluripotent stem cell-derived cardiomyocytes: electrophysiological properties of action potentials and ionic currents. Am J Physiol Heart Circ Physiol 301: H2006–H2017, 2011. 21890694 10.1152/ajpheart.00694.2011 PMC4116414

[3] Clark AP, Clerx M, Wei S, Lei CL, de Boer TP, Mirams GR, Christini DJ, Krogh-Madsen T. Leak current, even with gigaohm seals, can cause misinterpretation of stem cell-derived cardiomyocyte action potential recordings. Europace 25: 1532–2092, 2023. doi:10.1093/europace/euad243. PMC10445319 37552789

[4] Blinova K, Schocken D, Patel D, Daluwatte C, Vicente J, Wu JC, Strauss DG. Clinical trial in a dish: personalized stem cell-derived cardiomyocyte assay compared with clinical trial results for two QT-prolonging drugs. Clin Transl Sci 12: 687–697, 2019. doi:10.1111/cts.12674. 31328865 PMC6853144

[5] Blinova K, Dang Q, Millard D, Smith G, Pierson J, Guo L, Brock M, Lu HR, Kraushaar U, Zeng H, Shi H, Zhang X, Sawada K, Osada T, Kanda Y, Sekino Y, Pang L, Feaster TK, Kettenhofen R, Stockbridge N, Strauss DG, Gintant G. International multisite study of human-induced pluripotent stem cell-derived cardiomyocytes for drug proarrhythmic potential assessment. Cell Rep 24: 3582–3592, 2018. doi:10.1016/j.celrep.2018.08.079. 30257217 PMC6226030

[6] Clark AP, Wei S, Kalola D, Krogh-Madsen T, Christini DJ. An in silico-in vitro pipeline for drug cardiotoxicity screening identifies ionic pro-arrhythmia mechanisms. Br J Pharmacol 179: 4829–4843, 2022. doi:10.1111/bph.15915. 35781252 PMC9489646

[7] Kernik DC, Morotti S, Wu HDi, Garg P, Duff HJ, Kurokawa J, Jalife J, Wu JC, Grandi E, Clancy CE. A computational model of induced pluripotent stem-cell derived cardiomyocytes incorporating experimental variability from multiple data sources. J Physiol 597: 4533–4564, 2019. doi:10.1113/JP277724. 31278749 PMC6767694

[8] Paci M, Hyttinen J, Aalto-Setälä K, Severi S. Computational models of ventricular- and atrial-like human induced pluripotent stem cell derived cardiomyocytes. Ann Biomed Eng 41: 2334–2348, 2013. doi:10.1007/s10439-013-0833-3. 23722932

[9] Lei CL, Clerx M, Whittaker DG, Gavaghan DJ, de Boer TP, Mirams GR. Accounting for variability in ion current recordings using a mathematical model of artefacts in voltage-clamp experiments. Philos Trans A Math Phys Eng Sci 378: 20190348, 2020. doi:10.1098/rsta.2019.0348. 32448060 PMC7287334

[10] Lei CL. Model-Driven Design and Uncertainty Quantification for Cardiac Electrophysiology Experiments (PhD thesis). University of Oxford, 2020.

[11] Clerx M, Collins P, de Lange E, Volders PGA. Myokit: a simple interface to cardiac cellular electrophysiology. Prog Biophys Mol Biol 120: 100–114, 2016. doi:10.1016/j.pbiomolbio.2015.12.008. 26721671

[12] Virtanen P, Gommers R, Oliphant TE, Haberland M, Reddy T, Cournapeau D, , et al {SciPy} 1.0: fundamental algorithms for scientific computing in Python. Nat Methods 17: 261–272, 2020 [Erratum in Nat Methods 17: 352, 2020]. doi:10.1038/s41592-019-0686-2. 32015543 PMC7056644

[13] Feyen DAM, McKeithan WL, Bruyneel AAN, Spiering S, Hörmann L, Ulmer B, Zhang H, Briganti F, Schweizer M, Hegyi B, Liao Z, Pölönen R-P, Ginsburg KS, Lam CK, Serrano R, Wahlquist C, Kreymerman A, Vu M, Amatya PL, Behrens CS, Ranjbarvaziri S, Maas RGC, Greenhaw M, Bernstein D, Wu JC, Bers DM, Eschenhagen T, Metallo CM, Mercola M. Metabolic maturation media improve physiological function of human iPSC-derived cardiomyocytes. Cell Rep 32: 107925, 2020. doi:10.1016/j.celrep.2020.107925. 32697997 PMC7437654

[14] Banyasz T, Horvath B, Jian Z, Izu LT, Chen-Izu Y. Sequential dissection of multiple ionic currents in single cardiac myocytes under action potential-clamp. J Mol Cell Cardiol 50: 578–581, 2011. doi:10.1016/j.yjmcc.2010.12.020. 21215755 PMC3047417

[15] Lei CL, Wang K, Clerx M, Johnstone RH, Hortigon-Vinagre MP, Zamora V, Allan A, Smith GL, Gavaghan DJ, Mirams GR, Polonchuk L. Tailoring mathematical models to stem-cell derived cardiomyocyte lines can improve predictions of drug-induced changes to their electrophysiology. Front Physiol 8: 986–13, 2017. doi:10.3389/fphys.2017.00986. 29311950 PMC5732978

[16] Verkerk AO, Wilders R, Van Borren MM, Tan HL. Is sodium current present in human sinoatrial node cells? Int J Biol Sci 5: 201–204, 2009. doi:10.7150/ijbs.5.201. 19240810 PMC2646265

[17] Vinogradova TM, Zhou YY, Bogdanov KY, Yang D, Kuschel M, Cheng H, Xiao RP. Sinoatrial node pacemaker activity requires Ca(2+)/calmodulin-dependent protein kinase II activation. Circ Res 87: 760–767, 2000. doi:10.1161/01.res.87.9.760. 11055979

[18] Horváth A, Lemoine MD, Löser A, Mannhardt I, Flenner F, Uzun AU, Neuber C, Breckwoldt K, Hansen A, Girdauskas E, Reichenspurner H, Willems S, Jost N, Wettwer E, Eschenhagen T, Christ T. Low resting membrane potential and low inward rectifier potassium currents are not inherent features of hiPSC-derived cardiomyocytes. Stem Cell Reports 10: 822–833, 2018. doi:10.1016/j.stemcr.2018.01.012. 29429959 PMC5918194

[19] Van de Sande DV, Kopljar I, Maaike A, Teisman A, Gallacher DJ, Bart L, Snyders DJ, Leybaert L, Lu HR, Labro AJ. The resting membrane potential of hSC-CM in a syncytium is more hyperpolarized than that of isolated cells. Channels (Austin) 15: 239–252, 2021. doi:10.1080/19336950.2021.1871815. 33465001 PMC7817136

[20] Goversen B, Becker N, Stoelzle-Feix S, Obergrussberger A, Vos MA, van Veen TAB, Fertig N, de Boer TP. A hybrid model for safety pharmacology on an automated patch-clamp platform: Using dynamic clamp to join iPSC-derived cardiomyocytes and simulations of *I*_k1_ ion channels in real-time. Front Physiol 8: 1094–10, 2017. doi:10.3389/fphys.2017.01094. 29403387 PMC5782795

[21] Doss MX, Di Diego JM, Goodrow RJ, Wu Y, Cordeiro JM, Nesterenko VV, Barajas-Martínez H, Hu D, Urrutia J, Desai M, Treat JA, Sachinidis A, Antzelevitch C. Maximum diastolic potential of human induced pluripotent stem cell-derived cardiomyocytes depends critically on I_Kr_. PLoS One 7: e40288, 2012. doi:10.1371/journal.pone.0040288. 22815737 PMC3396384

[22] Es-Salah-Lamoureux Z, Jouni M, Malak OA, Belbachir N, Al Sayed ZR, Gandon-Renard M, Lamirault G, Gauthier C, Baro I, Charpentier F, Zibara K, Lemarchand P, Beaumelle B, Gaborit N, Loussouarn G. HIV-Tat induces a decrease in IKr and IKs via reduction in phosphatidylinositol-(4,5)-bisphosphate availability. J Mol Cell Cardiol 99: 1–13, 2016. doi:10.1016/j.yjmcc.2016.08.022. 27590098

